# Design and Analysis of an Extended Simply Supported Beam Piezoelectric Energy Harvester

**DOI:** 10.3390/s23135895

**Published:** 2023-06-25

**Authors:** Wei-Jiun Su, Chu-Hsiang Tseng

**Affiliations:** Department of Mechanical Engineering, National Taiwan University, No. 1, Sec. 4, Roosevelt Rd., Taipei 10617, Taiwan

**Keywords:** piezoelectric energy harvester, simply supported beam, strain distribution

## Abstract

The harvesting efficiency of a cantilevered piezoelectric energy harvester is limited by its uneven strain distribution. Moreover, a cantilevered harvester requires a large workspace due to the large displacement of its free end. To address these issues, a novel piezoelectric energy harvester based on an extended simply supported beam is proposed. The proposed design features a simply supported piezoelectric main beam with an extended beam attached to its roller end and a tip mass to reduce the resonant frequency. The theoretical model of the proposed piezoelectric energy harvester is developed based on the Euler–Bernoulli beam theory. The model has been experimentally validated through the fabrication of a prototype. The extended beam and tip mass are adjusted to see their influence on the performance of the harvester. The resonant frequency can be maintained by shortening the extended beam and increasing the tip mass simultaneously. A shorter extend beam leads to a more even strain distribution in the piezoelectric layer, resulting in an enhanced output voltage. Moreover, the simulation results show that a torsional spring is installed on the roller joint which greatly influences the voltage output. The strain distribution becomes more even when proper compressive preload is applied on the main beam. Experiments have shown that the proposed design enhances the output power by 86% and reduces tip displacement by 63.2% compared to a traditional cantilevered harvester.

## 1. Introduction

Piezoelectric energy harvesting has garnered significant attention for its potential in powering self-sustaining devices, wireless sensor networks, and wearable electronics. The conventional piezoelectric energy harvester (PEH) uses a cantilevered structure, which has been extensively studied for its performance. Although the cantilevered PEH offers a compact design, it has limitations such as a limited bandwidth and uneven distribution of strain across the piezoelectric material. These limitations impact the overall performance of cantilevered PEHs. To address the narrow bandwidth issue, multi-modal techniques [1,2,3,4,5,6,7,8,9] and nonlinear methods [10,11,12,13,14,15,16] have been utilized to increase the harvesting bandwidth.

On the other hand, the cantilevered structure’s uneven strain distribution limits its energy conversion efficiency. High strain at the fixed end can cause cracks in the piezoelectric material, while low strain at the free end results in low conversion efficiency. Additionally, the free end of a cantilevered PEH requires a large workspace to oscillate under excitations, particularly for low-frequency PEHs. Modifying the beam profile is a commonly used method to improve strain distribution. Roundy et al. [17] increased the power output of PEHs by utilizing triangular or trapezoidal beams. Goldschmidtboeing and Woias [18] developed a theoretical model for the triangular PEH and analyzed the effect of truncated ratio and tip mass on resonant frequency and power output. Benasciutti et al. [19] optimized the geometry of trapezoidal cantilevered PEHs to maximize power output. Dietl and Garcia [20] improved the beam profile to enhance both strain distribution and power output. A PEH with an optimal curved shape performs better than those with rectangular or linear tapered shapes. Reilly et al. [21] powered a wireless sensor node using a trapezoidal cantilevered PEH, which exhibits more evenly distributed strain across the piezoelectric plate. Modifying the beam profile is not the only solution. Paquin and St-Amant [22] proposed a cantilevered PEH with variable thickness to enhance the strain distribution, and Čeponis et al. [23] developed a trapezoidal cantilevered PEH with irregular cross-sections to further evenly distribute strain.

Improving strain distribution can also be achieved by modifying boundary or continuous conditions. Clamped-clamped beams have shown improved strain distribution and broadband harvesting capability [24,25]. A clamped-clamped buckled-beam PEH installed on flexible boundaries [26] expanded its output power and bandwidth. Li et al. [27,28] introduced revolute joints to the design of a cantilevered PEH to enhance the strain of the piezoelectric material, resulting in improved power output. Yang et al. [29] introduced an arc-shaped PEH, which exhibits improved stress distribution as well as power output to its cantilevered counterpart. Lu et al. [30] proposed a pin-based hybrid energy harvester combining a single-span buckled beam, a two-span buckled beam, and a two-span beam for broadband harvesting. Simply supported beams [31,32,33,34,35,36,37] are also commonly used and exhibit good strain distribution.

Force amplification mechanisms, which directly apply compression or tension to the piezoelectric materials to achieve even strain distribution, have been used to increase power output and broaden harvesting bandwidth. Xu et al. [38] integrated a force-amplified cymbal piezoelectric transducer to the design of a cantilevered PEH to achieve high power output at low frequency. Liu et al. [39,40,41] combined bi-stable structure and force amplification mechanisms to achieve high power output and broad bandwidth. Li et al. [42] proposed a piezoelectric-triboelectric energy harvester with a truss mechanism for force amplification and a stopper for broadening bandwidth. Yang et al. [43,44] attached a cymbal piezoelectric transducer to a pair of beams, allowing it to bear dynamic compressive loads under excitation. Unlike other studies that used force amplification mechanisms to compress piezoelectric materials, Chang and Su [45] proposed a tensile-mode PEH that utilizes a force-amplification mechanism to stretch a polyvinylidene fluoride (PVDF) film for energy generation. Force amplification mechanisms can improve energy harvesting performance by achieving an even strain distribution of the piezoelectric materials. However, implementing such mechanisms requires a large amount of space.

To improve the strain distribution of the piezoelectric material and consequently the power output, an extended simply supported beam (ESSB) PEH is proposed in this paper. This paper is structured as follows: Section 2 covers the design and modeling of the ESSB PEH. Section 3 depicts the prototype of the ESSB PEH and the experimental setup. The results of simulation and experiments of the ESSB PEH with different parameters are discussed in Section 4, and a comparison with a cantilevered PEH is made. Finally, the conclusion is drawn in Section 5.

## 2. Design and Modeling

**Figure 1:**
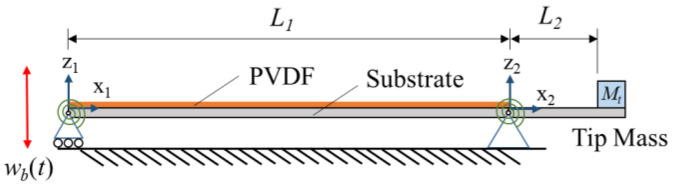
Schematics of the ESSB PEH.

The schematics of the proposed ESSB PEH are shown in Figure 1. The ESSB PEH consists of a main beam (*L*_1_), covered with a PVDF layer for energy generation, and an extended beam (*L*_2_) attached at the pin end. Torsional springs are attached to the roller and pin joints of the simply supported beam. A tip mass (*M_t_*) is also added to the free end of the extended beam for frequency adjustment.

The model of the proposed ESSB PEH is derived based on the Euler–Bernoulli beam theory. The PEH is modeled as a two-segment beam, and the equation of motion of each segment under free vibration can be written as:

(1) $$\{\begin{array}{l} YI_{1}\frac{\partial^{4}w_{r1}(x_{1},t)}{\partial x_{1}{}^{4}}-P\frac{\partial^{2}w_{r1}(x_{1},t)}{\partial x_{1}{}^{2}}+m_{1}\frac{\partial^{2}w_{r1}(x_{1},t)}{\partial t^{2}}=0\\ YI_{2}\frac{\partial^{4}w_{r2}(x_{2},t)}{\partial x_{2}{}^{4}}+m_{2}\frac{\partial^{2}w_{r2}(x_{2},t)}{\partial t^{2}}=0 \end{array}$$

where *YI_n_* is the bending stiffness; *P* is the axial compressive preload, which is below the critical load in this paper; *w* is the transverse displacement; *m_n_* is the mass density per unit length; the subscripts *r* and *n* indicate the mode and segment number, respectively. The transverse displacement can be expressed as:

(2) $$w_{rn}(x_{n},t)=\sum_{r=1}^{\infty }\phi_{rn}(x_{n})\eta_{r}(t),\;n=1,\;2$$

where *ϕ* and *η* are the mode shape and temporal function, respectively. The mode shape function can be expressed as:

(3) $$\{\begin{array}{l} \phi_{r1}(x_{1})=A_{r1}\cos(\lambda_{r11}x_{1})+B_{r1}\sin(\lambda_{r11}x_{1})+C_{r1}\cosh(\lambda_{r12}x_{1})+D_{r1}\sinh(\lambda_{r12}x_{1})\\ \phi_{r2}(x_{2})=A_{r2}\cos(\lambda_{r2}x_{2})+B_{r2}\sin(\lambda_{r2}x_{2})+C_{r2}\cosh(\lambda_{r2}x_{2})+D_{r2}\sinh(\lambda_{r2}x_{2}) \end{array}$$

where *λ_rn_* is the eigenvalue, which is a function of the undamped natural frequency *ω_r_*:

(4) $$\{\begin{array}{l} \lambda_{r11}=\sqrt{\frac{-P+\sqrt{P^{2}+4\omega_{r}{}^{2}YI_{1}m_{1}}}{2YI_{1}}}\\ \lambda_{r11}=\sqrt{\frac{P+\sqrt{P^{2}+4\omega_{r}{}^{2}YI_{1}m_{1}}}{2YI_{1}}}\\ \lambda_{r2}=\sqrt[{4}]{\frac{\omega_{r}{}^{2}m_{2}}{YI_{2}}} \end{array}$$

*A_rn_*, *B_rn_*, *C_rn_*, and *D_rn_* are the coefficients to be determined by the boundary and continuous conditions. The boundary and continuous conditions of the proposed PEH are presented in Equations (5)–(12).

(5) $$w_{r1}(0)=0$$

(6) $$YI_{1}{w^{\prime \prime }}_{r1}(0)-k_{1}{w^{\prime }}_{r1}(0)=0$$

(7) $$w_{r1}(L_{1})=0$$

(8) $$w_{r2}(0)=0$$

(9) $${w^{\prime }}_{r1}(L_{1})-{w^{\prime }}_{r2}(0)=0$$

(10) $$YI_{1}{w^{\prime \prime }}_{r1}(L_{1})-YI_{2}{w^{\prime \prime }}_{r2}(0)+k_{2}{w^{\prime }}_{r2}(0)=0$$

(11) $$YI_{2}{w^{\prime \prime }}_{r2}(L_{2})+I_{t}{\ddot{w^{\prime }}}_{r2}(L_{2})=0$$

(12) $$YI_{2}{w^{\prime \prime \prime }}_{r2}(L_{2})-M_{t}{\ddot{w}}_{r2}(L_{2})=0$$

where *I_t_* and *M_t_* are the moment of inertia and the mass of the tip mass, respectively; *k*_1_ and *k*_2_ are the stiffnesses of the torsional springs at the roller and the revolute joints, respectively. For the cases with no spring attached, simply set the stiffnesses to zero. The boundary and continuous conditions can be rewritten as the matrix form:

(13) $$M_{8\times 8}\left[\begin{array}{c} A_{r1}\\ \vdots \\ D_{r2} \end{array}\right]=\left[\begin{array}{c} 0\\ \vdots \\ 0 \end{array}\right]$$

where M is an 8-by-8 matrix. The resonant frequencies of the system can be calculated by setting the determinant of M to zero. Moreover, the mode shape function can be normalized via the orthogonal condition:

(14) $$\sum_{n=1}^{2}m_{n}\int_{0}^{L_{n}}\phi_{rn}(x_{n})\phi_{sn}(x_{n})+M_{t}\phi_{r2}(L_{2})\phi_{s2}(L_{2})+I_{t}{\phi^{\prime }}_{r2}(L_{2}){\phi^{\prime }}_{s2}(L_{2})=\delta_{rs}$$

**Figure 2:**
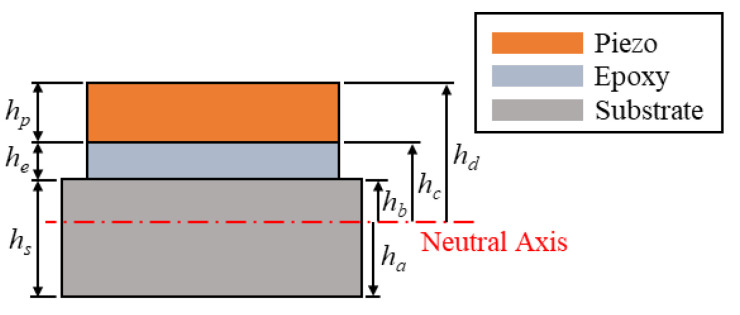
Cross-section view of the main beam.

The equation of motion of the system can finally be expressed as:

(15) $${\ddot{\eta }}_{r}(t)+2\zeta_{r}\omega_{r}{\dot{\eta }}_{r}(t)+\omega_{r}^{2}\eta_{r}(t)+Q_{r}v(t)=f(t)$$

where *ζ_r_* is the damping ratio; *Q_r_* is the electro-mechanical coupling coefficient:

(16) $$Q_{r}={\theta \frac{d\phi_{r1}(x_{1})}{dx_{1}}|}_{0}^{L_{1}},\;where\;\theta =-\frac{Y_{p}d_{31}b_{p}}{2h_{p}}(h_{d}^{2}-h_{c}^{2})$$

where *Y_p_*, *b_p_*, *h_p_*, and *d*_31_ are the Young’s modulus, width, thickness, and piezoelectric constant of the piezoelectric layer, respectively; *h_c_* and *h_d_* are the distances from the neutral axis of the composite beam to the bottom and top of the piezoelectric layer, respectively. The dimensions of the composite beam can be found in the cross-section view shown in Figure 2 The normalized external force *f* can be written as:

(17) $$f(t)=-\left[m_{1}\int_{0}^{L_{1}}\phi_{r1}(x_{1})dx_{1}+m_{2}\int_{0}^{L_{2}}\phi_{r2}(x_{2})dx_{2}+M_{t}\phi_{r2}(L_{2})\right]{\ddot{w}}_{b}$$

where *w_b_* is the displacement of the base excitation.

The PVDF layer is connected to a load resistor. The PVDF layer can be modeled as a current source in parallel with a capacitor. The current is a function of the temporal function. Therefore, the circuit can be expressed as:

(18) $$\frac{v(t)}{R}+C_{p}\frac{dv(t)}{dt}+\kappa_{r}{\dot{\eta }}_{r}=0$$

where *C_p_* is the capacitance of the PVDF layer and *κ_r_* is the modal coupling term and can be written as:

(19) $$\kappa_{r}=Y_{p}d_{31}b_{p}\frac{h_{d}^{2}-h_{d}^{2}}{2h_{p}}{\frac{d\phi_{r}(x)}{dx}|}_{0}^{L_{p}}$$

Combining Equations (15) and (18), the governing equations of the system are depicted as:

(20) $$\{\begin{array}{l} {\ddot{\eta }}_{r}(t)+2\zeta_{r}\omega_{r}{\dot{\eta }}_{r}(t)+\omega_{r}{}^{2}\eta_{r}(t)+Q_{r}v(t)=f(t)\\ \frac{v(t)}{R}+C_{p}\frac{dv(t)}{dt}+\kappa_{r}{\dot{\eta }}_{r}=0 \end{array}$$

The simulation is conducted in Matlab by using its built-in function ODE45 to solve the governing equations of the system.

## 3. Experiment

**Figure 3:**
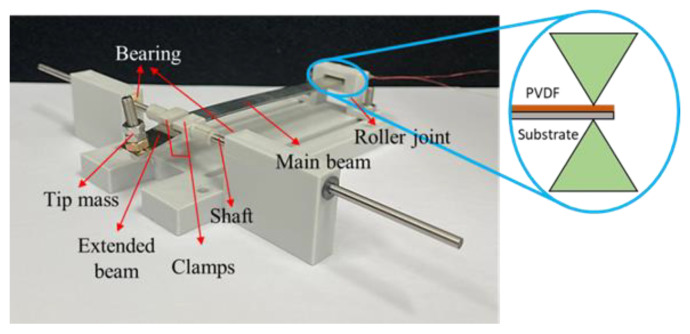
Prototype of the proposed ESSB PEH.

The prototype of the proposed ESSB PEH is depicted in Figure 3. The substrate of the beam is made of SUS301 stainless steel. A PVDF film (PolyK Technologies PVDF-P200-Al, State College, PA, USA) is attached to the substrate of the main beam by epoxy resin. A hole is drilled at the free end so a nut and a screw can be fixed and used as a tip mass. The extended beam and the main beam are connected at the pin support by a pair of clamps, which are attached to a pair of shafts. The shafts are inserted into sleeve bearings to form a revolute joint. The roller joint is realized by using a pair of wedges to constrain the transverse displacement of the beam as illustrated in Figure 3. The longitudinal displacement of the beam is allowed at the roller end.

**Figure 4:**
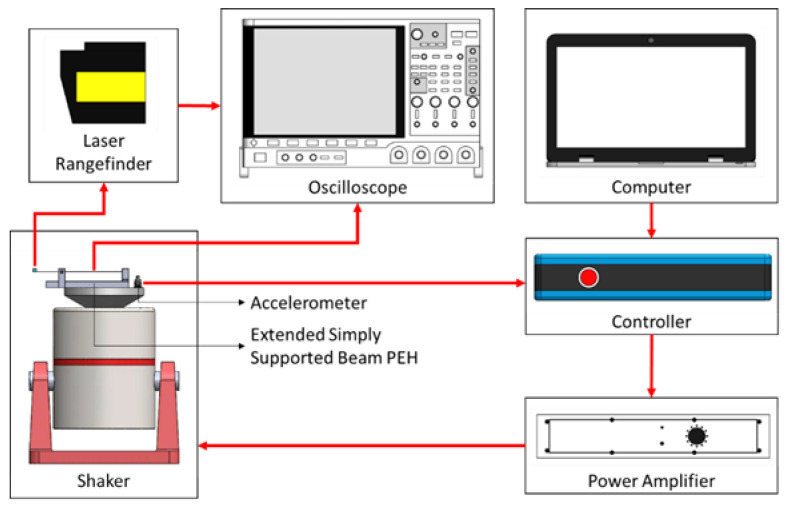
Schematics of the platform for base-excitation tests.

The schematics of the test platform are depicted in Figure 4. The PEH is mounted on a shaker (LDS V406, Nærum, Denmark) and tested under harmonic base excitations. The experiment is conducted using the vibration controller (ECON VT-9002, Hangzhou, China) to obtain the frequency responses through sweep sine tests. The sweep speed is limited to no more than 2.5 Hz/min to ensure steady-state responses. The voltage output and the tip displacement of the beam are measured by an oscilloscope (Keysight DSOX4042A, Santa Rosa, CA, USA) and a laser range finder (Mti LTS-120-40, Albany, NY, USA), respectively. The sampling frequency is 2000 samples/s. The capacitance of the PVDF film is measured by a multimeter (Rigol DM3058E, Suzhou, China). The damping ratio is obtained by fitting the simulated tip displacement to the experimental one.

## 4. Results

**Figure 5:**
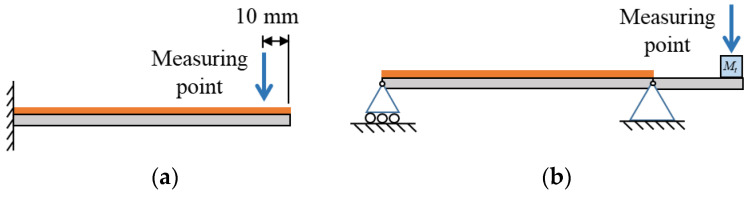
Illustration of the measuring points for displacement (**a**) cantilevered PEH (**b**) ESSB PEH.

In this section, the simulation results based on the theoretical model are validated with experimental results. Different lengths of the extended beam and tip masses will be tested to examine their influence on the performance of the ESSB PEH. The PEHs are tested under harmonic excitations with acceleration of 0.3 g. The performance of the proposed ESSB PEH will be compared with that of a cantilevered counterpart. It must be noted that the cantilevered PEH is identical to the main beam of the ESSB PEH to ensure a fair comparison between the two PEHs. The resonant frequency of the ESSB PEH is tuned to match that of the cantilevered PEH by adjusting the tip mass. The voltage output, strain distribution, and tip displacement will be investigated in the comparison. The measuring points of the PEHs for tip displacement are depicted in Figure 5. It can be seen that the measuring points are slightly in front of the free ends of the beams to prevent the laser from getting out of the range when the beams vibrate. The damping ratios of the PEHs are obtained by fitting the simulated and experimental displacements. The piezoelectric constant *d*_31_ is obtained by fitting the simulated and experimental voltages.

### 4.1. ESSB PEH under Base Excitations

**Figure 6:**
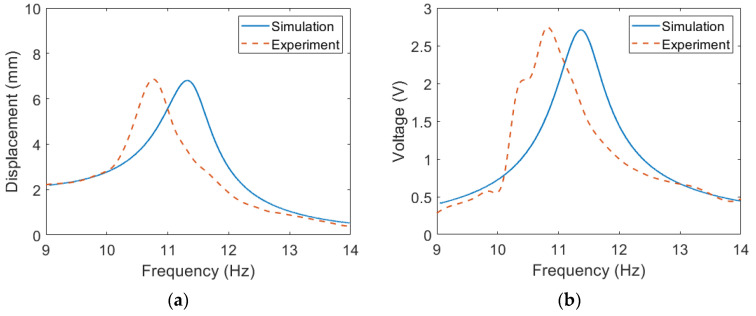

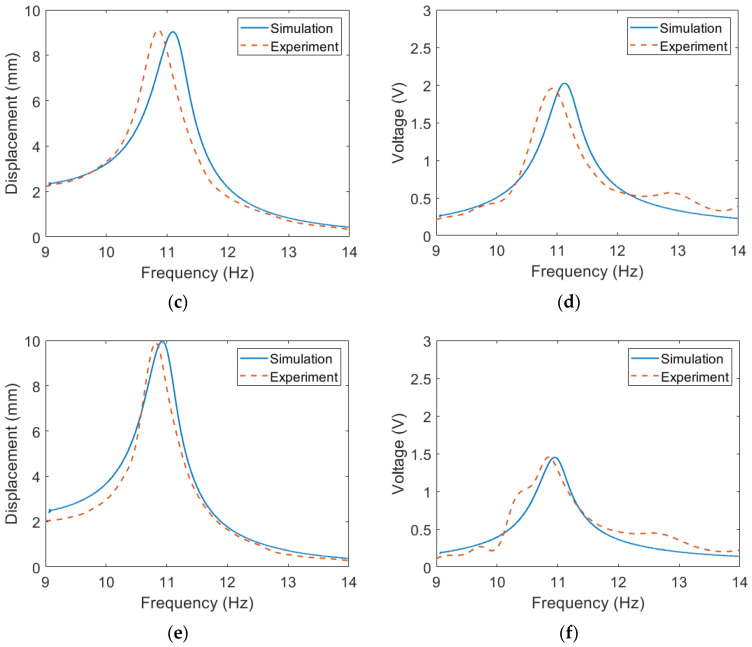
Simulation and experimental results of the proposed PEH with different *L*_2_ (**a**) Displacement, *L*_2_ = 20 mm, *M_t_* = 4.9 g (**b**) Voltage, *L*_2_ = 20 mm, *M_t_* = 4.9 g (**c**) Displacement, *L*_2_ = 30 mm, *M_t_* = 1.9 g (**d**) Voltage, *L*_2_ = 30 mm, *M_t_* = 1.9 g (**e**) Displacement, *L*_2_ = 40 mm, *M_t_* = 0.9 g (**f**) Voltage, *L*_2_ = 40 mm, *M_t_* = 0.9.

The parameters of the main beam and extended beam of the ESSB PEH are listed in Table 1, respectively. The displacement and voltage responses of the ESSB PEH with three different lengths of the extended beam are depicted in Figure 6. The damping ratios are obtained by fitting the simulated displacement response with the experimental one. The piezoelectric constant *d*_31_ is acquired by fitting to the experimental voltage. It is worth noting that each configuration of the extended beam has its own damping ratio, but all the configurations share the same piezoelectric constant.

The first resonant frequency of the ESSB PEH is tuned to 10.8 Hz for all the three configurations by adjusting the tip mass. For a shorter extended beam, a larger tip mass is required to achieve the same resonant frequency. It can be seen in Figure 6 that the simulation results match the experimental results well. The difference between the simulation and experimental resonant frequencies becomes more severe as the length of the extended beam decreases. This could be caused by the imperfect modeling of the tip mass. In this model, the tip mass is assumed to be a point mass. However, the tip mass used in the prototype is not a point mass, and therefore the error between the simulation and experimental results can be expected to increase as the tip mass becomes heavier. It is shown that the damping ratio decreases as the length of the extended beam increases and the tip mass decreases.

**Figure 7:**
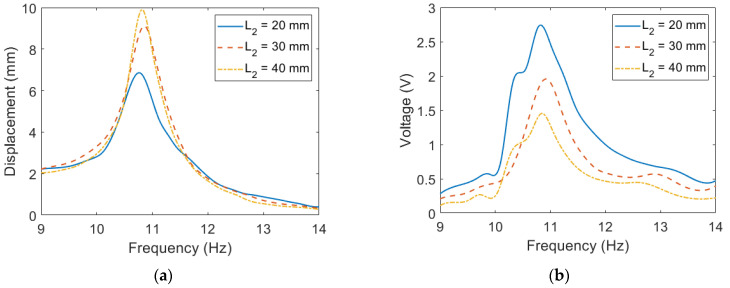
The experimental results of the proposed PEH with different *L*_2_ (**a**) Displacement (**b**) Voltage.

**Figure 8:**
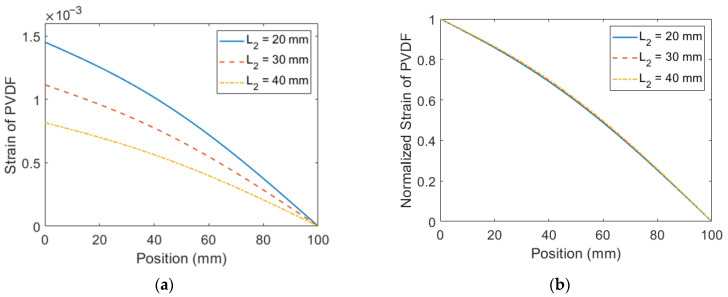
The strain distribution of PVDF on the proposed PEH with different *L*_2_ at its resonant frequency (**a**) theoretical strain distribution (**b**) theoretical normalized strain distribution.

The experimental displacement and voltage are further rearranged in Figure 7 for comparison among different configurations of the extended beam. The tip displacement of the configuration with the shortest extended beam is the lowest among the three configurations. This indicates that the workspace required for the configuration with the shortest extended beam is the smallest. On the other hand, even though a shorter extended beam leads to a higher damping ratio, it can be seen in Figure 7b that the configuration with the shortest extended beam exhibits the highest voltage output among the three configurations because its large mass leads to large excitation force. To exploit the cause of the performance difference among the three configurations, the strain of the PVDF is depicted in Figure 8. It is shown in Figure 8a that the configuration with the shortest extended beam has the largest strain, which is consistent with the voltage output. The normalized strain is depicted in Figure 8b. It is shown that the length of the extended beam does not influence the normalized strain distribution. Overall, shortening the length of the extended beam enhances the strain but has little impact on the normalized strain distribution.

### 4.2. ESSB PEH with Torsional Springs under Base Excitations

**Figure 9:**
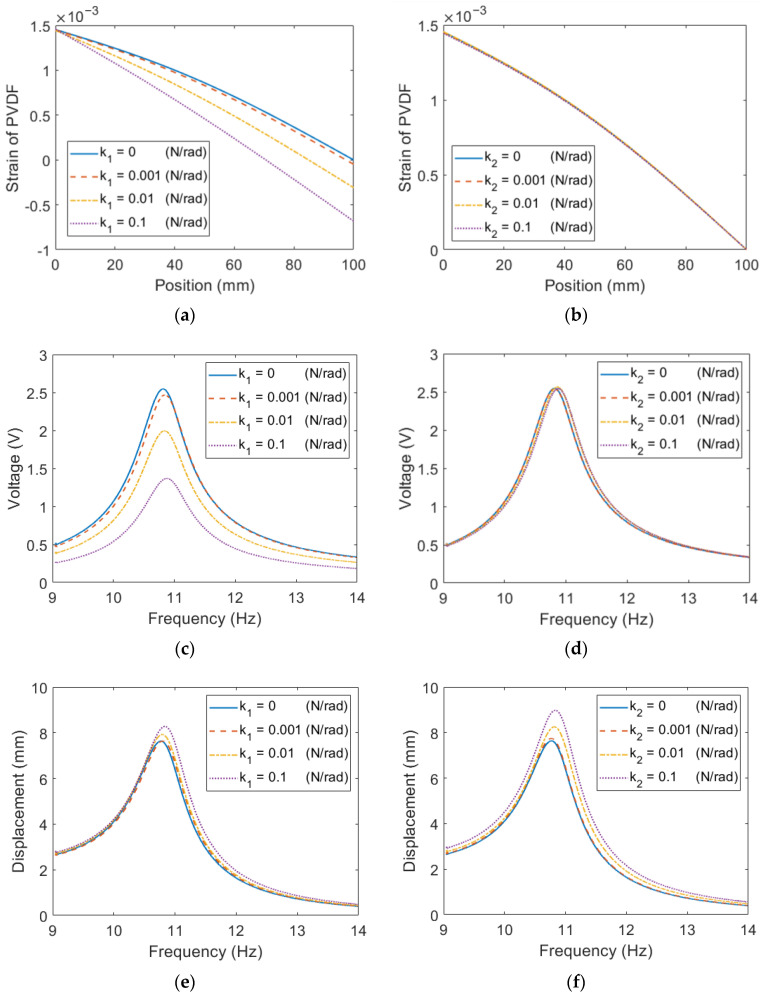
Simulation results of the ESSB PEH with torsional spring at the roller joint (**a**) Strain (**c**) Voltage (**e**) Displacement at the revolute joint (**b**) Strain (**d**) Voltage (**f**) Displacement.

This section examines the impact of boundary conditions on the performance of the proposed ESSB PEH. The influence is studied by installing a torsional spring on either the roller joint or the revolute joint. The parameters of each configuration are listed in Table 2 and Table 3. To ensure comparability, the resonant frequency is kept constant by adjusting the length of the extended beam and the tip mass. Figure 9 depicts the performance of the proposed ESSB PEH with a torsional spring. Results show that installing a torsional spring at the roller joint leads to an uneven strain distribution and decreased voltage output, as well as an increased tip displacement. However, when a torsional spring is installed at the revolute joint, the tip displacement increases, but no significant impact is observed in the voltage and strain distribution.

### 4.3. ESSB PEH with Axial Preload under Base Excitations

**Figure 10:**
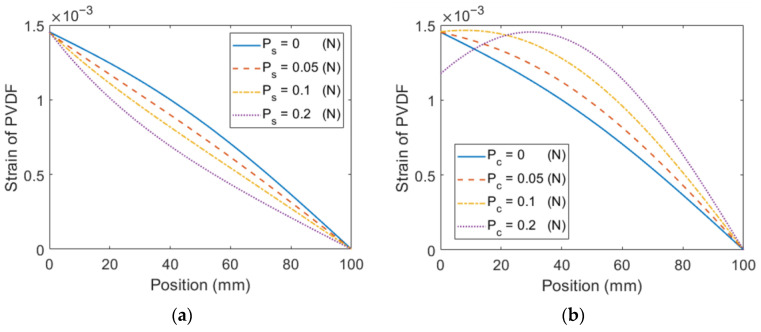

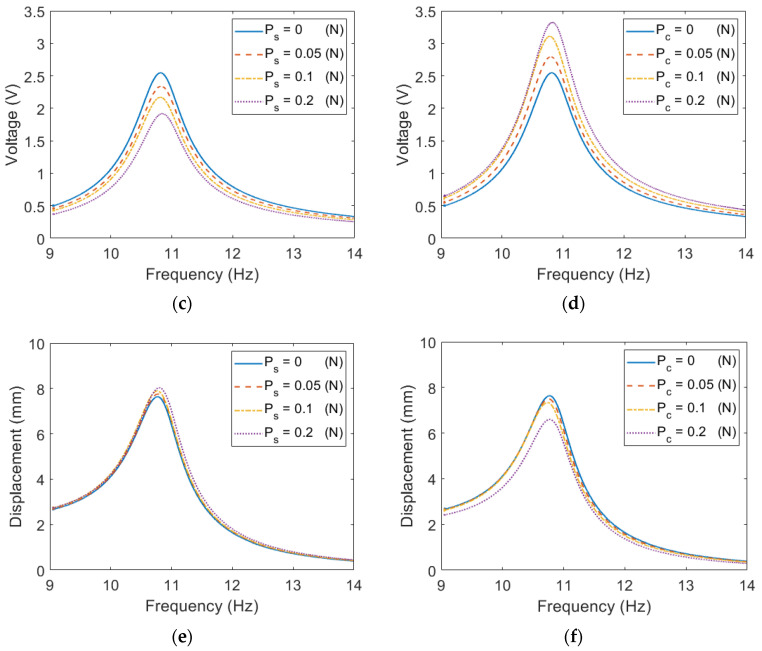
Simulation results of the ESSB PEH with axial preload stretching load (**a**) Strain (**c**) Voltage (**e**) Displacement compressing load (**b**) Strain (**d**) Voltage (**f**) Displacement.

The influence of the axial preload on the main beam is examined in this section. The parameters of the configurations with stretching and compressing loads are listed in Table 4 and Table 5, respectively. It is noted that the pre-compressing load is below the critical load. The resonant frequency is maintained by adjusting the length of the extended beam and the tip mass. It can be seen in Figure 10 that as the stretching load increases, the strain distribution becomes increasingly uneven, resulting in a diminished voltage output and an increased tip displacement. Conversely, the compressing load promotes a more even strain distribution, leading to an enhanced voltage output and a decreased tip displacement.

### 4.4. Comparison between the ESSB PEH and the Cantilevered PEH

**Figure 11:**
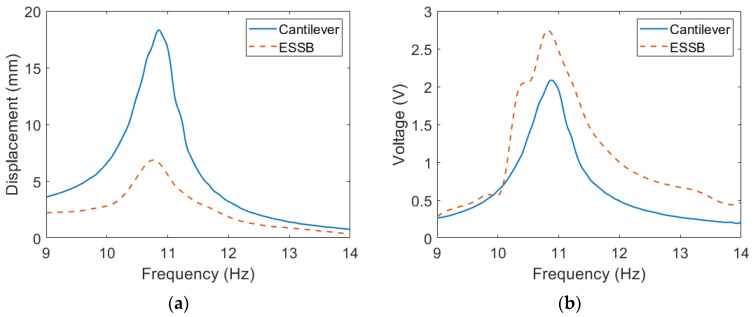
Experimental results of the ESSB PEH and a cantilevered PEH (**a**) Displacement (**b**) Voltage.

**Figure 12:**
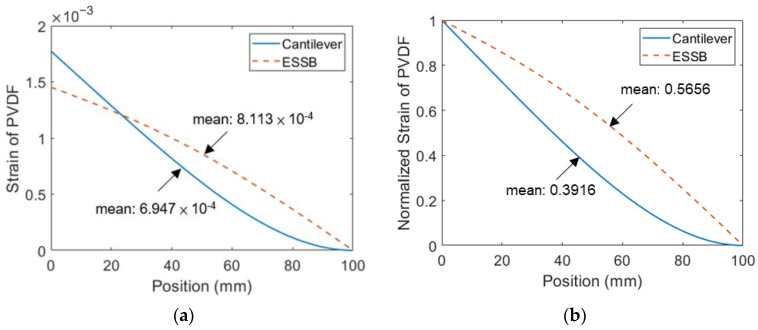
The strain distribution of PVDF on the ESSB PEH and a cantilevered PEH at their resonant frequencies (**a**) theoretical strain distribution (**b**) theoretical normalized strain distribution.

In this section, a comparison between the ESSB PEH with the extended beam of 20 mm and a cantilevered PEH is drawn to understand the advantages of the ESSB PEH. The experimental voltage and displacement are illustrated in Figure 11. The results show that the ESSB PEH has higher voltage output and lower tip displacement than the cantilevered counterpart. The voltage output of the ESSB PEH is 1.35 times that of the cantilevered PEH at their resonant frequencies. The tip displacement of the ESSB PEH is about one-third of that of the cantilevered PEH. The tip displacement of the ESSB PEH is greatly reduced when compared with the cantilevered PEH, which means that the proposed design requires a smaller workspace. The strain distributions of the two PEHs are revealed in Figure 12. It is shown that the maximum strain of the cantilevered PEH is larger than that of the ESSB PEH. However, the mean strain of the ESSB PEH is 44% higher than its cantilevered counterpart, which indicates that the ESSB PEH is more efficient than its counterpart. Figure 12b clearly demonstrates that the proposed ESSB PEH has a more evenly distributed strain than the cantilevered counterpart. Despite having a smaller maximum strain than the cantilevered design, the proposed ESSB design still generates higher output voltage.

**Figure 13:**
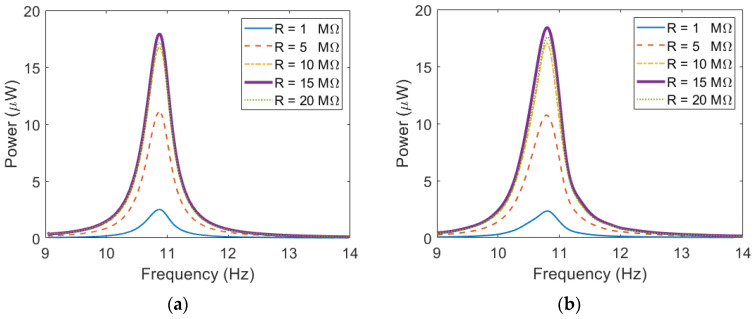

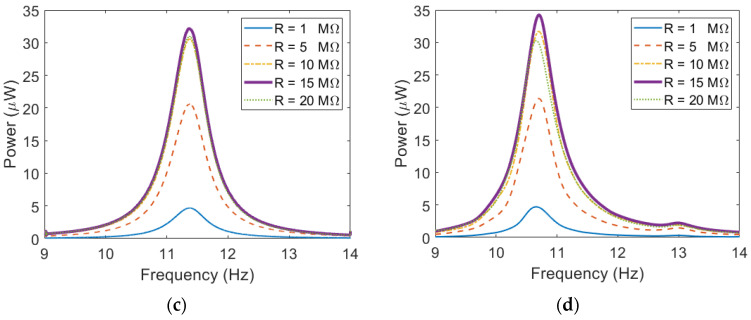
Power output with different load resistance (**a**) Cantilevered PEH—simulation (**b**) Cantilevered PEH—experiment (**c**) ESSB PEH—simulation (**d**) ESSB PEH—experiment.

Figure 13 shows the power outputs of the cantilevered PEH and the ESSB PEH with different load resistances. Both PEHs are examined under excitations of 0.3 g. The simulation results are consistent with the experimental results. It is shown that both PEHs achieve their highest output in both simulation and experimental results when the load resistance is 15 MΩ. In the experiment, the maximum power output of the ESSB PEH is 34.21 μW, which is higher than that of the cantilevered PEH by 86%. The optimal resistance can be calculated by:

(21) $$R_{opt}\approx \frac{1}{\omega_{r}C_{p}}$$

Based on Equation (21), the optimal resistance is about 14.7 MΩ, which is in accordance with results shown in Figure 13.

### 4.5. Influence of Tip Mass on Strain Distribution of the ESSB PEH

**Figure 14:**
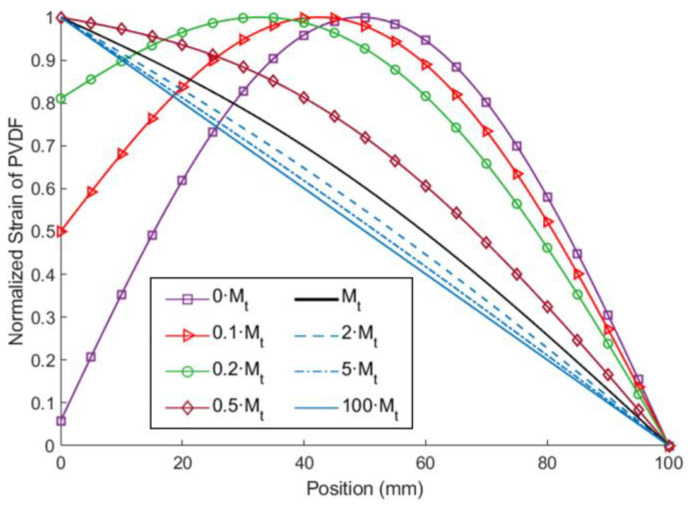
The theoretical normalized strain distribution of PVDF on the ESSB PEH with different tip masses.

The extended beam differs the ESSB structure from a simply supported beam structure. As the tip mass and the length of the extended beam is reaching zero, the ESSB structure will behave more like a simply supported beam structure. In this section, the tip mass is adjusted while the other parameters are fixed to see the influence of the tip mass on the strain distribution of the ESSB PEH. The parameters of the extended beam are indicated in configuration 1 of Table 1. Figure 14 depicted the strain distribution of the PVDF on the proposed PEH and a cantilevered counterpart. It can be seen that the strain distribution is nearly symmetric about the middle of the beam when the tip mass is zero. As the tip mass increases, the location of the maximum strain moves toward the roller joint, eventually resulting in a nearly straight-line strain distribution. To further quantify the strain distribution, the means of the normalized strain of each setting are calculated and listed in Table 6. Among the eight settings, the setting of 0.2·*Mt* shows the highest mean of the normalized strain. If the mass is further increased, the mean of the normalized strain will decrease, which means the strain distribution becomes more uneven.

## 5. Conclusions

This study proposes a novel ESSB PEH based on a simply supported beam with an extended beam attached to its pin end. The extended beam enhances the moment acting on the main beam for energy generation and also reduces the resonant frequency of the system. The theoretical model of the ESSB PEH is established based on the Euler–Bernoulli beam theory and validated with experiments. Three different settings of the extended beam and tip mass with the same resonant frequency are examined and compared. It is shown that the setting with the shortest extended beam exhibits the highest voltage output. The normalized strain distribution shows no significant difference among these three settings. When compared with a cantilevered PEH, the proposed PEH demonstrates better strain distribution and higher voltage output. The experimental results show that the proposed ESSB PEH outperforms its cantilevered counterpart by 86% in terms of power output. Finally, the tip mass shows a significant impact on the strain distribution. The strain distribution can be optimized by properly adjusting the tip mass.

## Figures and Tables

**Table 1:** Parameters of the extended beam.

Symbol	Description	Value		
		Config. 1	Config. 2	Config. 3
*L*_2_	Length of the extended beam	20 mm	30 mm	40 mm
*M_t_*	Tip mass	4.9 g	1.9 g	0.9 g
*ζ*	damping ratio	0.035	0.0265	0.0245
*b_x_*	Width of the extended beam	12.7 mm		
*h_x_*	Thickness of the extended beam	0.1 mm		
*ρ_x_*	Density of the extended beam	7930 kg/m^3^		
*Y_x_*	Young’s modulus of the extended beam	193 GPa		
*L*_1_	Length of the main beam	100 mm		
*b_s_*	Width of the substrate	12.7 mm		
*h_s_*	Thickness of the substrate	0.1 mm		
*ρ_s_*	Density of the substrate	7930 kg/m^3^		
*Y_s_*	Young’s modulus of the substrate	193 Gpa		
*b_e_*	Width of the epoxy	10 mm		
*h_e_*	Thickness of the epoxy	0.09 mm		
*ρ_e_*	Density of the epoxy	1200 kg/m^3^		
*Y_e_*	Young’s modulus of the epoxy	27 Mpa		
*b_p_*	Width of the PVDF	10 mm		
*h_p_*	Thickness of the PVDF	0.2 mm		
*ρ_p_*	Density of the PVDF	1780 kg/m^3^		
*Y_p_*	Young’s modulus of the PVDF	2.9 Gpa		
*C_p_*	Capacitance of the PVDF	1 nF		
*d*_31_	Piezoelectric constant of the PVDF	16 pm/V		

**Table 2:** Parameters of the ESSB PEH with a torsional spring at the roller joint.

Symbol	Description	Value			
		Config. A	Config. B	Config. C	Config. D
*k*_1_	Stiffness	0 N/rad	10^−3^ N/rad	10^−2^ N/rad	10^−1^ N/rad
*L_e_*	Length of the extended beam	21.9 mm	22.1 mm	23.9 mm	26.2 mm
*M_t_*	Tip mass	4.4 g	4.33 g	3.83 g	3.33 g

**Table 3:** Parameters of the ESSB PEH with a torsional spring at the revolute joint.

Symbol	Description	Value			
		Config. A	Config. B	Config. C	Config. D
*k*_1_	Stiffness	0 N/rad	10^−3^ N/rad	10^−2^ N/rad	10^−1^ N/rad
*L_e_*	Length of the extended beam	21.9 mm	21.8 mm	20 mm	12.2 mm
*M_t_*	Tip mass	4.4 g	4.64 g	7.4 g	50.6 g

**Table 4:** Parameters of the ESSB PEH with pre-stretching load at the main beam.

Symbol	Description	Value			
		Config. A	Config. B	Config. C	Config. D
*P_s_*	Pre-stretching load	0 N	0.05 N	0.1 N	0.2 N
*L_e_*	Length of the extended beam	21.9 mm	22.9 mm	23.8 mm	25.1 mm
*M_t_*	Tip mass	4.4 g	4.12 g	3.9 g	3.61 g

**Table 5:** Parameters of the ESSB PEH with pre-compressing load at the main beam.

Symbol	Description	Value			
		Config. A	Config. B	Config. C	Config. D
*k*_1_	Pre-compressing load	0 N	0.05 N	0.1 N	0.2 N
*L_e_*	Length of the extended beam	21.9 mm	20.8 mm	19.5 mm	18.5 mm
*M_t_*	Tip mass	4.4 g	4.74 g	5.2 g	4.9 g

**Table 6:** Means of the normalized strain.

Tip Mass	Mean of Normalized Strain
0·Mt	0.6465
0.1·Mt	0.7146
0.2·Mt	0.7379
0.5·Mt	0.6405
Mt	0.5656
2·Mt	0.5316
5·Mt	0.5124
100·Mt	0.5006

## Data Availability

The data presented in this study are available on request from the corresponding author.
